# The effects of beef protein isolate and whey protein isolate supplementation on lean mass and strength in resistance trained individuals - a double blind, placebo controlled study

**DOI:** 10.1186/1550-2783-12-S1-P11

**Published:** 2015-09-21

**Authors:** Matthew Sharp, Kevin Shields, Ryan Lowery, Jason Lane, Jeremy Partl, Chase Holmer, Julie Minevich, Eduardo De Souza, Jacob Wilson

**Affiliations:** 1 The University of Tampa, Tampa, FL, USA

## Background

Consumption of moderate amounts of whey and animal derived protein has been demonstrated to enhance short and long-term protein balance over a placebo matched control. However, to date no study has comprehensively compared high quality beef based protein supplementation with whey based protein sources following a resistance training protocol. The purpose of this study was to determine the effects of post-exercise consumption of two servings of beef protein isolate (BeefISO) or whey, compared to a maltodextrin control on lean mass and strength during 8 weeks of resistance training.

## Methods

Thirty college-aged, resistance-trained males and females were randomly assigned to one of three treatment groups. Subjects consumed two servings (46g) of Beef Protein Isolate (BeefISO™), Whey Protein isolate or maltodextrin. Subjects trained 5 days per week (3 resistance training, 2 cardio) for 8 weeks as a part of a daily undulating periodized resistance-training program. Two servings of protein were consumed immediately following exercise or at a similar time of day on off days. Dual emission x-ray absorptiometry (DXA) was used to determine changes in body composition. Maximum strength were assessed by one-rep-max (1RM) for bench press (upper body) and deadlift (lower body). A two-way ANOVA with repeated measures model was used to identify group, time, and group by time interactions. The significance level was set at p < 0.05.

## Results

Both beef protein isolate (↑5.7%) and whey protein isolate (↑4.7%) each lead to a significant increase in lean body mass compared with baseline (p < 0.0001). Fat loss was also significantly decreased at 8 weeks compared to baseline for beef protein isolate and whey, 10.8% and 8.3% respectively (p < 0.0001). 1RM both deadlift and bench-press were both significantly increased for all treatment groups when compared to baseline. However, no significant differences in increased strength as measured by deadlift (↑11.6%-19.3 %) or bench-press (↑11.4%-17.6%) were observed between beef protein isolate, whey, or maltodextrin groups over the 8 week training regimen (p < 0.0001).

## Conclusion

The results of this study further support the benefits of protein supplementation following resistance training. Specifically, in this study consumption of two-servings of beef protein isolate or whey resulted in significant gains in lean body mass over time, which outpaced gains resultant from resistance training alone (maltodextrin supplementation). However, all experimental groups increased strength equally. It is plausible that the uniform strength gains were explained by both increases in neural and morphological adaptations negating the effect of protein supplementation. Overall, the results of this study demonstrate that consuming two servings of either beef protein isolate or whey protein isolate following resistance training lead to significant increases in lean body mass and strength.

